# Street Tree Planning to Improve Public Health and Ecosystem Resilience in Urban Areas: A Scenario Analysis Using a System Dynamics Model

**DOI:** 10.3390/ijerph19031625

**Published:** 2022-01-31

**Authors:** Youngsun Seok, Hyosook Yim, Taehyeon Moon, Jinhyung Chon

**Affiliations:** 1 Department of Environmental Science and Ecological Engineering, Korea University, Seoul 02841, Korea; suni87@korea.ac.kr (Y.S.); am113noon@korea.ac.kr (T.M.); 2 BK21 FOUR Research & Education Center for Environmental Science and Ecological Engineering, Korea University, Seoul 02841, Korea; hsyim@korea.ac.kr; 3 Division of Environmental and Ecological Engineering, Korea University, Seoul 02841, Korea

**Keywords:** urban ecosystem health, urban air pollution, particulate matter, fine dust, species diversity, environmental benefits

## Abstract

Increases in particulate matter in cities threaten both public health and ecosystems. Street trees, which are a corridor-type green infrastructure capable of absorbing particulate matter, have been promoted as one possible solution to this problem. However, planting selected trees solely with the goal of reducing particulate matter may adversely affect street tree ecosystem resilience by inhibiting species diversity. This study aims to investigate urban street tree planting strategies that reduce particulate matter while maintaining ecosystem resilience. To this end, a study site in Suwon, South Korea was selected, and street tree planting scenarios were developed based on the selected site information. A scenario analysis was conducted using a system dynamics model. The model simulated the long-term trends under each scenario regarding the amount of particulate matter absorbed by the trees and the changes in species diversity. The analysis results clearly show that strategic planting of street trees while focusing on only a specific purpose—reducing particulate matter—can adversely affect ecosystem resilience. The scenario analysis also revealed that increasing the number of street trees while maintaining a balance among various species is the best option for reducing particulate matter without degrading species diversity. Additionally, the results support the need to plant evergreen species to consider the winter season.

## 1. Introduction

The World Health Organization (WHO) designated particulate matter (PM) (small particles floating or scattered in the atmosphere) as a first-class carcinogen in 2013. Previous studies reported that long-term, high concentrations of PM can cause cardiovascular and respiratory diseases, resulting in increased mortality [1,2,3,4]. Children, the elderly, and respiratory patients with even brief exposure times to PM or to a low concentration of PM are at increased risk of mortality [5,6,7]. Accordingly, PM pollution is considered one of the critical factors threatening public health.

Since cities are densely populated and feature heavy traffic, urban residents have a greater risk of PM exposure. In particular, the exposure risk in winter is more severe than in summer because heating systems produce more PM, and air circulation is poor [8,9]. Researchers in the environmental and landscaping fields have recently emphasized the importance of using green space to reduce PM in cities [10,11,12,13]. One proposed solution is to employ “green corridors”, a concept that includes street trees, wall greening, rooftop greening, green curtains, and green belts [14,15,16,17,18,19]. Among these, street trees have been highlighted as an important means of reducing PM [20]: they occupy less space than do parks and forests, which require substantial space; they reduce mobile pollutants and scattered dust, which are the main culprits of PM; and they contribute to creating a pleasant environment for pedestrians and community corridors. Planting a single street tree requires only an initial three-year maintenance fee (approximately $250–600); however, it has been reported that a total of more than $90,000 in direct benefits can be expected, excluding the aesthetic, social, and natural benefits that accrue over a tree’s life [20,21,22]. In other words, planting and managing street trees can generate high profits at low cost and respond to environmental issues such as reducing PM while improving urban ecosystem health.

When developing a street tree plan, determining which tree species to include is a complex issue, because the selection must match the various purposes and interests pursued by stakeholders, which include local governments, residents, and tree managers [23]. To effectively reduce PM, which has emerged as a serious environmental and social issue, it is necessary to actively adopt street tree species that have an excellent ability to absorb PM. Adopting a single street tree species can be more efficient both in terms of cost, such as initial purchase cost, and in terms of PM reduction. However, single-species selection could result in a rapid decrease in street tree species diversity and ecosystem resilience. Street tree clusters with low biodiversity indices are subject to pests and diseases and are more likely to fail to adapt to sudden environmental changes such as climate change [24,25,26]. Humans have already experienced the types of problems that emerge from mass tree death due to single species unification in the United States and Germany during the 1930s and 1980s, where *Ulmus davidiana* var. *japonica*, which was widely planted as a street tree in the United States, died collectively due to fungi introduced from the Netherlands in the 1930s. Moreover, collective tree death occurred in Germany in the 1980s due to acid rain. At that time, street tree species were selected with a focus on specific trees with high stress and pest resistance. As a result, some species of street trees were heavily overplanted, making them vulnerable as a group to pests [27,28,29]. These examples show the importance of careful planning before planting street trees so that the result maintains the diversity of street tree species and does not degrade ecosystem resilience.

This study aims to find strategies for urban street tree planting that both reduce PM pollution and maintain street tree ecosystem resilience by ensuring species diversity. We selected Suwon in South Korea as a study area involving a city which is striving to reduce the level of fine dust by establishing a comprehensive PM management plan for the 2021–2025 period [30]. We present street tree planting scenarios within the defined specific site in Suwon and evaluate the scenarios through system dynamics model (SD model) simulations developed for this study. The objectives of this work are as follows: First, we present the street tree planting scenarios for the study site. Second, we developed an SD model that can predict long-term developments in PM absorption by street trees while ensuring the species diversity of street trees. Third, we simulated the scenarios and evaluated the results with regard to both PM absorption and ensuring species diversity. Fourth, we also test the performances of the scenarios by focusing on the winter season when PM problems typically become more serious. The results of this study contribute to planning for street tree planting in urban areas by providing tree selection and quantity management strategies.

## 2. Materials and Methods

### 2.1. The Study Site

**Figure 1:**
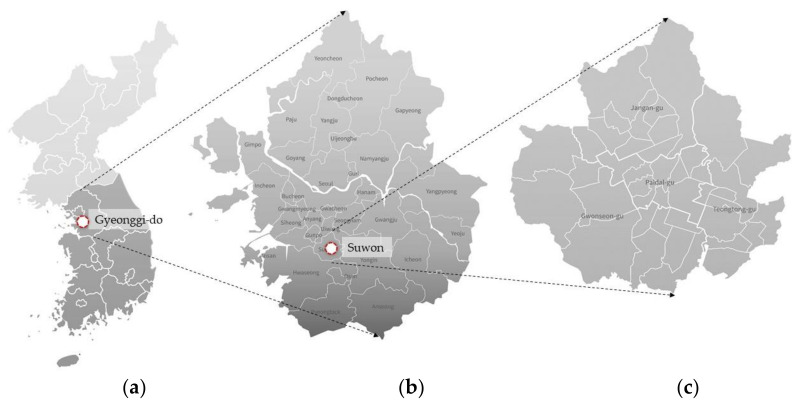
Study site: (**a**) South Korea; (**b**) Gyeonggi-do; (**c**) Suwon.

For this study, we selected a specific study site within Suwon (37°15′44″ N 127°01′44″ E) in Gyeonggi-do, South Korea (Figure 1). Suwon is a large South Korean city and is quite densely populated [31]. To determine the study site, we analyzed areas particularly vulnerable to PM in Suwon using ArcGIS. Variables determined based on previous studies were used to identify sites vulnerable to PM [32,33] and include the concentration of PM in the region, the density of PM emission facilities, the distribution status of people most vulnerable to PM (0–13 years old, 65 years of age or older), facility density (e.g., schools, hospitals, senior citizens, and others), and distance from a road.

**Figure 2:**
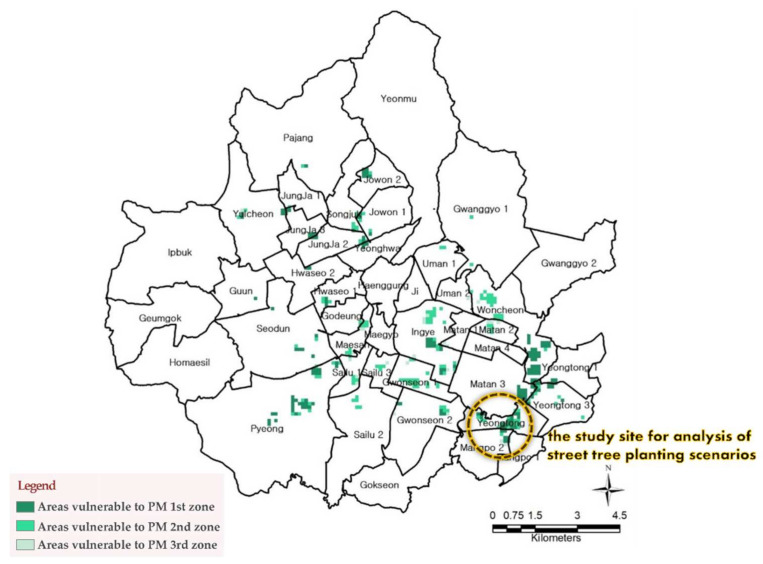
Results of a spatial analysis to identify areas vulnerable to PM in Suwon.

The variables were reclassified into three stages using a fuzzy overlay. The analysis identified the three stages (1–3) of zone areas most vulnerable to PM (Figure 2). Zone 1 indicates the areas most threatened by PM; these are areas where PM management is urgent. Thus, we searched for a study site within the first zone and surveyed places where the population is highly mobile. Additionally, we investigated multi-land roads where the sidewalks are sufficiently wide to accommodate various street tree planting scenarios. We used satellite maps and field surveys for this investigation. Finally, we selected the study site, an 1822 m street road near Mangpo Station, located in Yeongtong-gu, Suwon with an area of 27.67 km^2^. This area includes eight lane roads, subways, bus stops, and shopping malls and is widely used by people who are vulnerable to PM.

**Figure 3:**
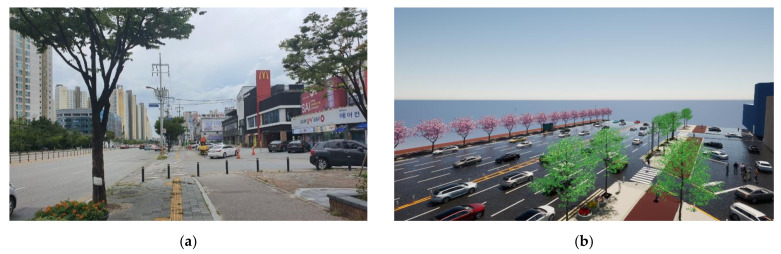
Status of the study site: (**a**) a picture of the study site; (**b**) a 3D image of the study site.

The data for the study site were obtained from official government statistics [34]. The study site includes 208 street trees and includes a variety of species, including 112 *Zelkova serrata*, 70 *Prunus yedoensis*, and 26 other tree species. The area with a sidewalk width of 5 m or more is extended for approximately 711 m, and it is legally possible to plant double rows of trees here. The length of the road is 1271 m (driveways were excluded from the total road distance and calculations were performed only on the area where street trees can be planted). The planting distance between the street trees in the study site is 8 m or more on average. Based on this information, we developed street tree planting scenarios. Figure 3 shows the status of the study site visualized using Twinmotion Educational ver. 2021.1.4.

### 2.2. System Dynamics Model

We adopted a system dynamics model (SD model) to analyze the street tree planting scenarios. The SD model is a simulation methodology that can codify the interrelationships of different variables into a model and predict the changes to each variable over time through computer simulation. The SD model enables the future outcomes resulting from previous decisions to be assessed [35,36]. Scenario analyses using an SD model have been used in previous ecological landscape studies [11,37,38].

#### 2.2.1. Purpose of the Model

The SD model developed in this study is targeted at supporting the process of planning street tree planting. The model monitors the changes in PM absorption and species diversity of street trees over time based on a given street tree plan. Each of the scenarios developed for this study differed in species selection strategy and total number of trees. The future outcomes derived from the developed scenarios are evaluated based on the SD model simulations with the goal of discovering a plan that increases street tree PM absorption but does not degrade species diversity.

#### 2.2.2. Model Description

**Figure 4:**
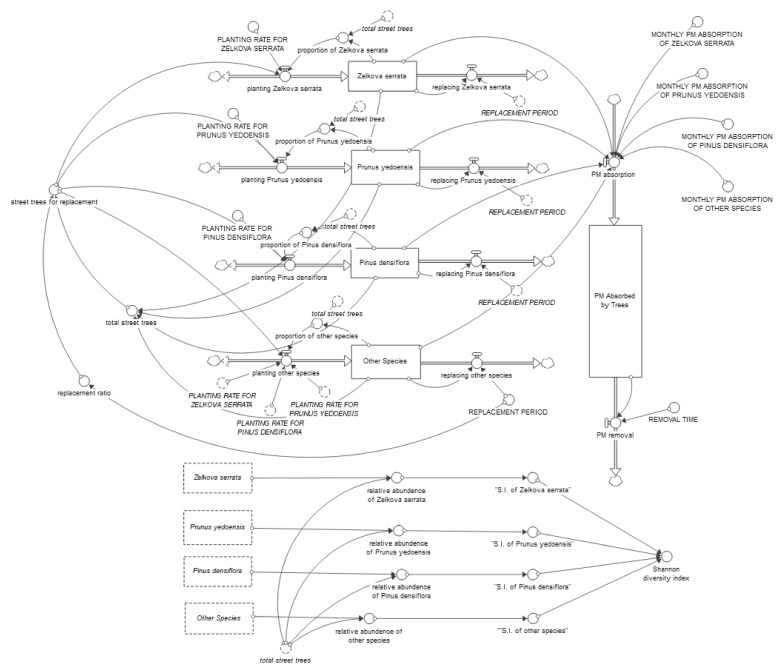
SD model’s stock-flow diagram.

Figure 4 shows a stock-flow diagram of the SD model built in this study. In this figure, the forms of the boxes in the figure represent tree stocks whose characteristics accumulate or diminish over time. Variables such as tree populations and PM absorption correspond to the stocks. The double-line arrows with valves indicate flow variables, while the circles refer to the variables or to the parameters of converters [39,40].

Each tree in the model is replaced with a new tree when a prespecified replacement period elapses. Without a policy intervention, trees are replaced by the same species. However, when a policy intervention occurs to plant a specific species B as a priority, the existing species A will be replaced by species B. The planting rate per month of species A is calculated by the following equations:

When no species has a priority for the replacement:

Planting A = the number of street trees for replacement × proportion of A(1)

When species B has a priority for replacement:

Planting A = the number of street trees for replacement × (1 − targeted ratio for planting B) × proportion of A without B(2)

Equation (1) shows the situation in which species A is replaced by species A, while Equation (2) indicates the case where the target ratio of species B has priority; then, only the remaining quantity is replaced with species A. These conditional equations determine the number of each type of tree planted at each point in time. The dynamics of the tree species distribution rely on the accumulating changes in this process over time. Meanwhile, the distribution of tree species at any one point in time determines the amount of PM absorbed by the street trees at the study site. PM absorption by street trees can be expressed by the following equation:

(3) $$MPA=\sum \left(Tree\times PmA\right),$$

where *MPA* refers to the total monthly PM absorption of street trees. *PmA* represents the average monthly absorption of each species. The sum of the data for each species is the *MPA*. The amount of PM absorption by street trees changes in conjunction with the change in the tree species distribution. The changes in species diversity by tree species were measured using the Shannon diversity index, which is a comprehensive measurement of species evenness and diversity that is generally used as a measure of species diversity in clusters in ecology [41].

After building the SD model structure, data were collected to determine the input values for each parameter. The street tree replacement cycle was assumed to be 20 years. The *PmA* (average monthly PM absorption of each tree type) data were derived from the values of previous studies. After estimating the input values for the parameters, the model equations were entered into the model to maintain unit consistency. The model simulation used STELLA Professional ver. 2.0. The simulation period was set from 2021 to 2050; the time unit of the simulation was monthly. The entire model structure and the input values of the variables are included in Supplementary Materials (Table S1).

### 2.3. Estimation of Monthly PM Absorption by Each Species of Tree

Few prior studies have estimated the value of the average annual PM absorption by trees in South Korea. In 2017, the National Institute of Forest Science, under direction of the Korea Forest Service, measured the PM concentrations in urban forests and downtown areas. They suggested an estimate that the PM absorbed per tree is approximately 35.7 g per year [42]. Since then, local governments in South Korea have used these data to establish urban forest construction plans and determine urban forest sizes [43,44]. However, the amount of PM absorbed by trees can differ depending on each tree’s diameter at breast height (DBH), leaf area, and leaf pore size. Additionally, tree PM absorption can be affected by weather and land-use conditions [45,46]. Therefore, accurately estimating the amount of PM absorption for each tree is complex.

In 2018, the Seoul Institute reported data on the amount of PM absorption by trees in urban areas. This study analyzed the amount of PM absorption for *Ginkgo biloba*, *Platanus occidentalis*, *Zelkova serrata*, *Prunus yedoensis*, and *Pinus densiflora*, which are urban street trees widely used in Seoul, South Korea. The researchers obtained leaf samples through fieldwork. They analyzed leaf samples from trees in urban forests and street trees and estimated the absorption amount of PM per unit area of the leaves (mg/m^2^) [47,48,49]. Then, they calculated the PM absorption by multiplying the values by the leaf area index (LAI). As a result, the Seoul Institute announced the annual estimated absorption of PM as 99.4 g/tree (*Platanus occidentalis)*, 66.6 g/tree (*Zelkova serrata)*, 45.3 g/tree (*Prunus yedoensis)*, 24.2 g/tree (*Pinus densiflora)*, and 10.7 g/tree (*Ginkgo biloba)* [10].

This study uses the PM absorption data by tree species from the study conducted by the Seoul Institute in 2018 for the model input values. This was deemed acceptable because Suwon, the study site for this study, and Seoul have similar geographical, social, economic, and environmental characteristics; thus, the amount of PM absorption by trees is expected to be similar. However, of these trees, *Platanus occidentalis* often causes pollen allergy problems and *Ginkgo biloba* absorbs a relatively small amount of PM and frequently elicits civil complaints caused by the smell of the ginkgo nuts. Therefore, this study excluded *Platanus occidentalis* and *Ginkgo biloba* from the street tree planting scenarios. The remaining tree species (*Zelkova serrata*, *Prunus yedoensis*, and *Pinus densiflora)* were included in the model.

Table 1 shows the annual average PM absorption for each tree species used in this study. Compared to other species, whose average absorption is approximately 35.7 g of PM per year, *Zelkova serrata* reaches 66.6 g, and *Prunus yedoensis* averages 45.3 g, which are larger. *Pinus densiflora* has a smaller value compared to the others, with an annual average of 24.2 g, but because it is an evergreen tree, it can be considered first when focusing on reducing PM in winter.

### 2.4. Estimation of Seasonal PM Absorption by Each Species of Tree

PM pollution is known to have a seasonal cycle. Based on a time series analysis of PM concentration, the PM concentration level is highest in March and lowest in August [50]. PM pollution tends to be more severe in winter than in summer because heating systems release more PM-causative substances and because NO_3_ discharged from automobile exhaust gas in cold temperatures disperses poorly and remains stable in the atmosphere for an extended period. Recent studies conducted in South Korea also mentioned the importance of selecting measures for winter control that consider seasonal PM pollution cycles [51]. Therefore, this study estimates the seasonal variation in PM absorption by trees and assesses their effect.

**Figure 5:**
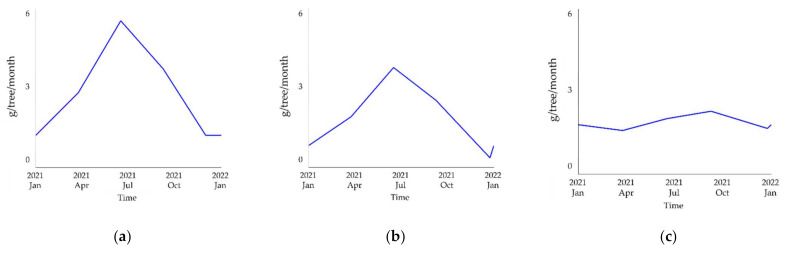
Variation graphs of monthly PM absorption by tree species: (**a**) *Zelkova serrata*; (**b**) *Prunus yedoensis*; (**c**) *Pinus densiflora*.

A tree’s ability to absorb PM is heavily affected by variations in the seasonal leaf area of the tree. We constructed a graph to display the variation in the seasonal amount of PM absorption by each species following the method suggested by 2017 research of Verryckt et al. [52]. First, the variation pattern in the seasonal leaf area index was estimated for each species. Then, a graph showing the variation in the seasonal amount of PM absorption by each species was produced from the pattern in the seasonal leaf area index. The seasonal data on the leaf area index of deciduous and coniferous forests were obtained from [53]. Additionally, data on the leaf area index of pine forests were obtained from [54]. The estimation results are shown in Figure 5.

### 2.5. Street Tree Planting Scenarios at the Selected Site

Table 2 presents the details of the street tree planting scenarios developed in this study. The baseline scenario indicates the status of street trees in the selected study site. The other eight scenarios compare PM absorption and tree species diversity with the baseline scenario. The scenarios have two categories: ‘Replace-only scenarios’ and ‘Additional Tree Planting scenarios’. The first category includes the Replace-only scenarios; these consist of plans that simply replace tree species but do not change the total number of street trees. The total number of street trees was fixed at 208 in these scenarios. Depending on which species to plant first when replacing, the scenarios include Rep_only_Zelko (intensive replacement with *Zelkova serrata*), Rep_only_Prun (intensive replacement with *Prunus yedoensis*), Rep_only_Pinus (intensive replacement with *Pinus densiflora*), and Rep_only_Mix (mixed replacement with *Zelkova serrata*, *Prunus yedoensis*, and *Pinus densiflora)*. In these scenarios, the target intensive planting ratio of the designated species is set to 70% but adjusted upward to 90% to observe the changes based on the planting ratio intensity.

The second category involves the Additional Tree Planting scenarios, in which the distance between street trees is minimized to achieve the maximum number of street trees at the selected site. According to the guidelines and regulations on the design and management of street trees published by the Korea Forest Service and the local government in Suwon [55,56,57], the distance between street trees must be at least 6 m. In addition, if the width of the sidewalk is greater than 5 m, double row planting is possible. Another recommendation is to define tree planting areas in road medians to reduce PM. Following these guidelines and regulations, we established Additional Planting Scenarios.

**Figure 6:**
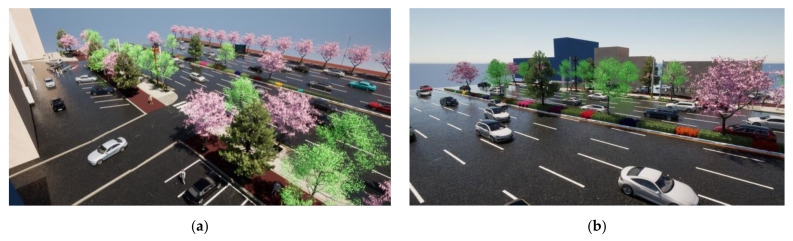
3D landscape images of (**a**) street trees planted in double rows; (**b**) street trees planted on the road median.

The Additional Tree Planting Scenarios adopted double row planting in sections exceeding 5 m in width of the road around Mangpo Station and included plans to create green space in the road median as well as to minimize street tree spacing. Landscape images showing double row planting and road median planting areas are displayed in Figure 6.

Consequently, the Additional Tree Planting scenarios increased the total street tree population to 388 trees (208 existing trees and 180 additional trees). There were four scenarios, including Plant_more_Zelko (all additional trees were *Zelkova serrata*), Plant_more_Prun (all additional trees were *Prunus yedoensis*), Plant_more_Pinus (all additional trees were *Pinus densiflora*), and Plant_more_Mix (added trees were evenly divided among *Zelkova serrata*, *Prunus yedoensis*, and *Pinus densiflora*). The intensive ratio for the species was set to 70%.

**Figure 7:**
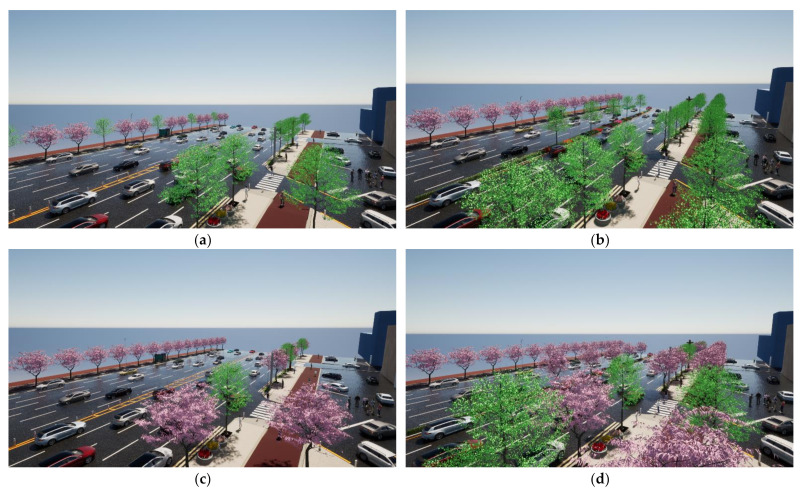

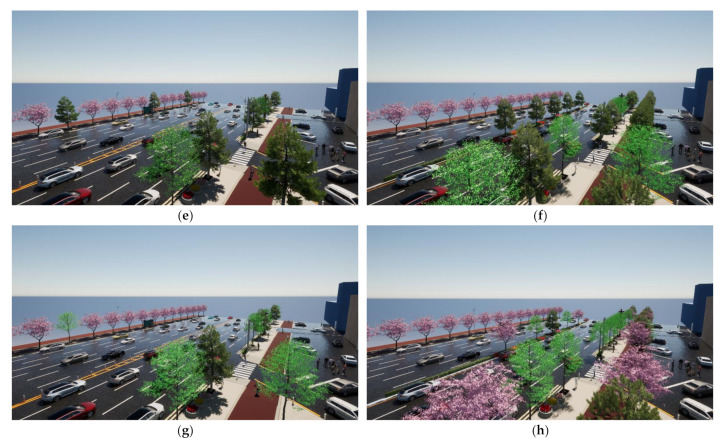
3D landscape images for each scenario: (**a**) Rep_only_Zelko; (**b**) Plant_more_Zelko; (**c**) Rep_only_Prun; (**d**) Plant_more_Prun; (**e**) Rep_only_Pinus; (**f**) Plant_more_Pinus; (**g**) Rep_only_Mix; (**h**) Plant_more_Mix.

The input values for each scenario are shown in Table 2. Figure 7 shows a 3D image of the expected landscape for each scenario.

## 3. Results

### 3.1. Replace-Only Scenarios

**Figure 8:**
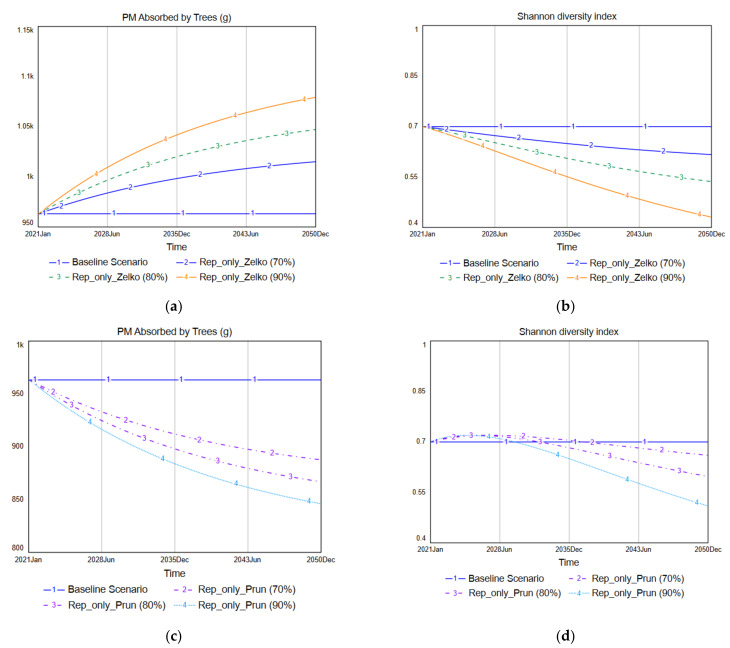

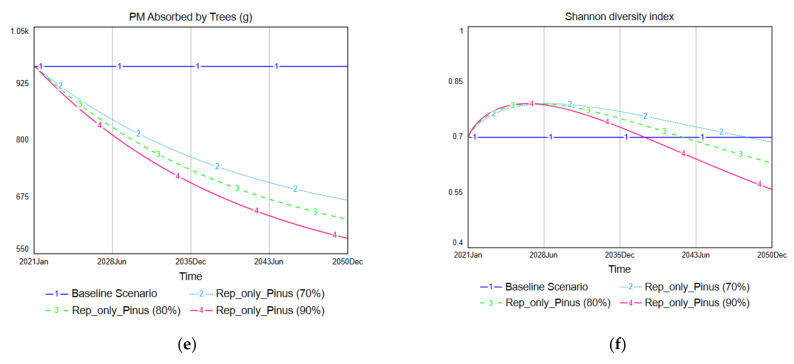
Simulation results of the Replace-only scenarios: (**a**) PM absorption of Rep_only_Zelko; (**b**) Species diversity of Rep_only_Zelko; (**c**) PM absorption of Rep_only_Prun; (**d**) Species diversity of Rep_only_Prun; (**e**) PM absorption of Rep_only_Pinus; (**f**) Species diversity of Rep_only_Pinus.

The Replace-only scenarios are those in which some of the existing species are replaced with new species, but the total number of trees planted in the selected study site is maintained. Figure 8 shows the simulation results for the Rep_only_Zelko, Rep_only_Prun, and Rep_only_Pinus scenarios (which are Replace-only scenarios). The simulated PM absorption results reveal that, among these three scenarios, Rep_only_Zelko is the only scenario in which the PM absorption of street trees increases compared with the baseline scenario. The baseline scenario has an average PM absorption of 963 g/month, and Rep_only_Zelko increases that to 1080 g/month. In contrast, Rep_only_Prun reduces the monthly average PM absorption to 846 g/month, while Rep_only_Pinus reduces it to 583 g/month.

The simulations of the Shannon diversity index shown in Figure 8 reveal that Rep_only_Zelko threatens the species diversity of street trees the most. The baseline scenario retains the Shannon diversity index at 0.7, but Rep_only_Zelko eventually reduces it to 0.43. This outcome occurs because *Zelkova serrata* was the dominant species in the initial distribution of the tree species. In contrast, Rep_only_Prun and Rep_only_Pinus increased the species diversity over the short term because *Prunus yedoensis* was not the dominant initial species and *Pinus densiflora* did not previously exist at the site. Rep_only_Pinus results in a 13% increase in street tree species diversity. However, both scenarios also decrease the species diversity over the long term.

**Figure 9:**
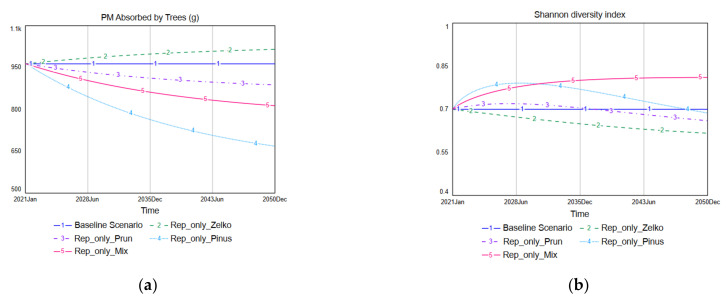
Comparison of the Replace-only scenarios: (**a**) PM absorption; (**b**) Species diversity.

Figure 9 compares the performances of three scenarios (Rep_only_Zelko, Rep_only_Prun, Rep_only_Pinus) to Rep_only_Mix. Rep_only_Mix results in the best species diversity performance, but it decreases the total amount of PM absorption by trees, reducing the monthly average PM absorption amount from 963 g/month to 813 g/month while improving the Shannon diversity index from 0.7 to 0.8. No scenario improved both PM absorption and species diversity in the Replace-only scenarios. These results indicate that there is a tradeoff between PM absorption by trees and species diversity. In other words, when one of these two performance indicators improves, the other shows a pattern of deterioration.

### 3.2. Additional Tree Planting Scenarios

**Figure 10:**
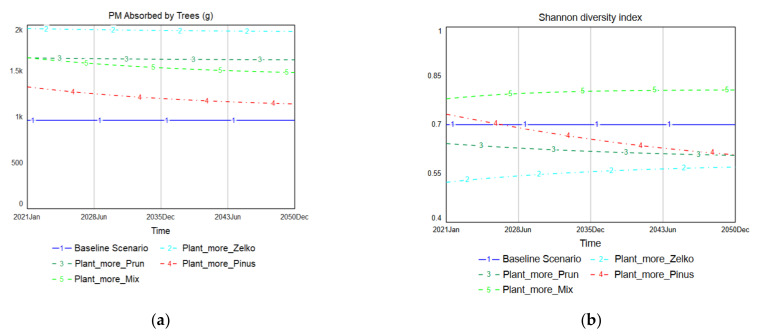
Comparison of Additional Tree Planting scenarios: (**a**) PM absorption amount; (**b**) species diversity.

The Additional Tree Planting scenarios were designed to increase tree planting to the maximum number possible at the selected site. Consequently, under these scenarios, 180 more trees were planted than under the Replace-only scenarios described in Section 3.1. Figure 10 presents the simulation results of the Additional Tree Planting scenarios. Since the total number of trees increased, all the Additional Tree Planting scenarios exhibit higher levels of PM absorption than does the baseline scenario. The Plant_more_Zelko scenario results in the highest level of PM absorption, reaching an average of 1960 g/month. Plant_more_Prun maintains the baseline level around an average of 1620 g/month. The performance of Plant_more_Pinus changes from an average of 1330 g/month in 2021 to 1140 g/month in 2050, which exceeds the baseline scenario. Plant_more_Mix begins at 1640 g/month in 2021 and decreases to 1480 g/month by 2050, but is still above the level of the baseline scenario.

In terms of species diversity, Plant_more_Mix showed the best performance. The baseline scenario preserves the Shannon diversity index at 0.7, while Plant_more_Mix remains at approximately 0.8. Plant_more_Pinus initially improves the Shannon diversity index but ultimately decreases over time until it reaches the 0.6 level. Plant_more_Prun and Plant_more_Zelko both reduced species diversity compared to the baseline scenario.

**Figure 11:**
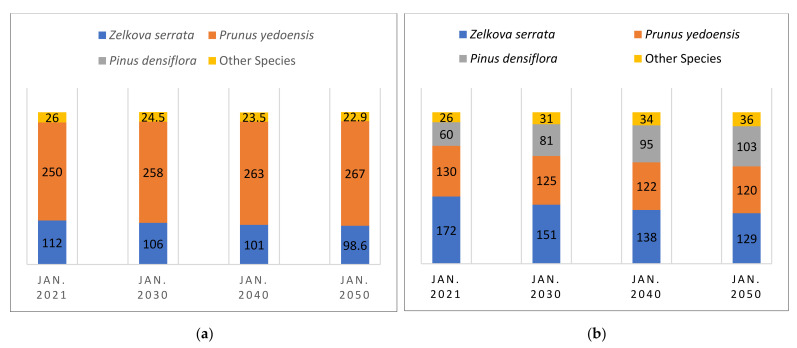
Distribution changes in tree species over time: (**a**) Plant_more_Prun; (**b**) Plant_more_Mix.

As shown in Figure 10, Plant_more_Mix is the only scenario that reaches a higher level of PM absorption than the baseline scenario while also improving species diversity. This implies that when planting more street trees with a mixture of various trees, it is possible to not only increase PM absorption by street trees but also enhance species diversity. Plant_more_Prun produces a similar or a higher level of PM absorption than Plant_more_Mix but shows contradictory results in its species diversity trend. Both scenarios reach almost the same level of PM absorption in January 2021, with an average of 1643–1644 g/month. However, the Shannon diversity index of Plant_more_Prun remains below that of the baseline scenario. This is due to changes in the tree species distribution. As shown in Figure 11, Plant_more_Prun becomes more biased toward the specific species over time through the intensive planting of *Prunus yedoensis*, while the tree species in the Plant_more_Mix scenario become more evenly distributed over time.

### 3.3. Scenario Analysis When Considering Seasonal Changes in Tree PM Absorption

Leaves play a crucial role in the PM absorption mechanism of street trees [49] because the amount of PM absorption is affected by the changes in a tree’s leaf area. This study performed a scenario analysis using the seasonal trend estimations for the PM absorption of each species, as shown in Figure 5. Since PM problems in winter are more severe than those in summer, this analysis focused on the trees’ ability to absorb PM in winter.

**Figure 12:**
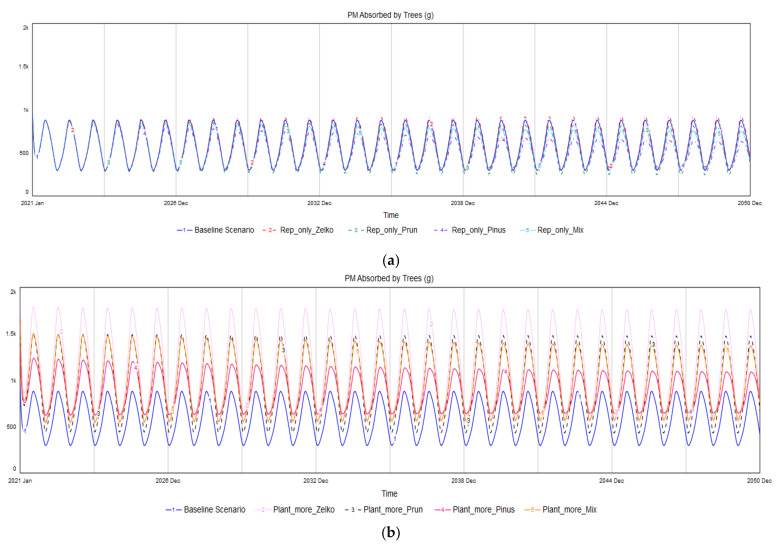
Scenario simulations reflecting the seasonal changes in PM absorption by trees: (**a**) replace-only scenarios; (**b**) Additional Tree Planting scenarios.

Figure 12 displays the analysis results. Of the replace-only scenarios, Plant_more_Pinus is the only one in which PM absorption in winter improved compared with the baseline scenario. However, the degree of improvement is quite small. In contrast, all the Additional Tree Planting scenarios achieved higher PM absorption values in winter than that of the baseline scenario. Among them, Plant_more_Pinus exhibits the highest PM absorption in winter, followed by Plant_more_Mix and then Plant_More_Zelko.

### 3.4. The Comparison of Scenario Analysis Results

Table 3 summarizes the results of the scenario analysis. Different criteria may apply for the scenario assessments. When the goal is to reach decisions that maximize the PM absorption ability of street trees, Plant_more_Zelko (the Additional Tree Planting scenario concentrating on *Zelkova serrata*) was revealed as the best choice. However, Plant_more_Prun and Plant_more_Mix could also be good choices to achieve that goal. In contrast, when the goal is to reach decisions which increase the species diversity of street trees, Rep_only_Mix is the best option, followed by Plant_more_Mix and then Rep_only_Pinus as the second and third alternatives in terms of producing desirable results.

Plant_more_Mix, a scenario in which more trees are planted by harmonizing several species, turned out to be the only option that can both increase PM absorption by trees and improve species diversity as well. However, if a decision-maker has the goal of prioritizing PM absorption in winter, Plant_more_Pinus will be the best alternative, and Plant_more_Mix can be an additional option aimed at achieving desirable results with respect to reducing PM levels in winter.

## 4. Discussion

This study established a variety of street tree planting scenarios at sites vulnerable to PM in Suwon, South Korea and then simulated the planting scenarios using a system dynamics model projected until 2050. These simulations reflect future changes in PM absorption by street trees under the various scenarios. Additionally, they showed the tree species diversity trends under the scenarios. Finally, we assessed the planting scenarios based on the simulation outcomes.

The results of this study made clear that street tree planning based on a single criterion of “PM absorption” or “species diversity” is apt to face an undesirable outcome. This is because a tradeoff relationship exists between these two criteria. Efforts to increase the amount of PM absorption by street trees tend to reduce tree species diversity. Therefore, street tree planners, including city planners, landscape architects, ecologists, civil servants, etc., should pursue harmony with these two aspects when arriving at their decisions. More generally, the result implies that the planning and management of street trees should be undertaken with an approach from multiple perspectives rather than a single goal. Focusing on a single objective such as reducing PM pollution by street trees revealed risks involving the loss of other valuable environmental benefits.

Although this study solely focused on two different benefits, street trees offer various benefits, such as sequestering carbon, reducing the urban heat-island effect, elevating the livability of cities, etc. Understanding the interrelationships and dynamics of the various benefits of street trees is necessary to purse harmony, but it is a highly complex matter. Therefore, future research will need to provide further knowledge and a range of decision support tools purposed to help planners harmonize the different aspects of street tree planting so as to meet the different goals and needs of street tree planting.

The SD model developed in this study can be a tool for developing strategies for tree species selection as well as their mixtures. The model prospects the future outcome of species diversity and PM absorption by street trees under the different street tree planting scenarios. Even though this study had selected a study site and conducted the scenario analysis, the SD model can apply to another geographical location as long as there is PM absorption data for each tree species at the site.

This study has presented a level of guideline knowledge for establishing a street tree planting strategy by comparing each scenario under different criteria (see Table 3). Depending on the difference in the social demands or urgency of any inherent issues, the priority of criteria can be varied in reality. For example, maximizing PM absorption by trees may be the top priority goal when planting street trees in an area where there is generally high vehicle traffic and a lot of socially and physically vulnerable groups. By contrast, maximizing species diversity of street trees may be the urgent goal in areas where evidence of ecosystem vulnerability due to the unification of species is being reported. In these cases, increasing the number of street trees with a unified species or only replacing tree species without changing the number of trees can be the suitable solution. However, even in such cases, it would be desirable to consider the fact that pursuing only a single goal risks losing other valuable benefits.

The mixed planting strategy was the best strategy for street tree planting for the goal of overcoming the tradeoff between PM uptake and species diversity, both worthy planning achievements. Previous studies argued that when selecting a tree species to maximize urban forest health, the ratio of a single species should be under 10%, the ratio of the same genus should be within 20%, and the ratio of the same family should be within 30% [58]. In addition, some argue that these mixed planting standards should be applied to street tree planting in Korea [59]. The results of this study support previous researchers’ claims that it is necessary to set standards for mixed planting and enforce them bindingly. Additionally, considering the problem of PM in winter, planting evergreen species such as *Pinus densiflora* is a way to reduce the high PM concentrations in winter. This result supports the conclusions of previous studies that evergreen species of street trees should be planted at a certain minimum level to consider the winter season [10,60,61,62].

## 5. Conclusions

Planting street trees is a potential solution for reducing PM pollution in cities in light of their function as a source of PM absorption. This study conducted scenario analyses so as to investigate various street tree planning strategies for urban street tree planting which both alleviate PM pollution and protect the species diversity of street trees.

First, we developed street tree planting scenarios at a specific study site in Suwon, South Korea. The site showed high vulnerability to PM pollution with the potential to threaten the health of urban residents. Second, we constructed an SD model for the scenario analysis. The model simulations allowed for envisioning the future development in PM absorption by street trees and the species diversity among them under each planting scenario. Finally, we discussed the results of the scenario analysis to discern a more general understanding of the plans for street tree plantings.

The scenario analyses clearly showed that there is a tradeoff relationship between PM absorption by trees and their species diversity. This means that seeking a single goal for planting street trees, such as absorbing PM to improve public health, will lead to a degradation in ecosystem diversity in the long term. The effort of harmonization among the different goals and needs for street trees is necessary to escape degradation in terms of ecosystem resilience.

In addition, based on the scenario analysis, we reached the conclusion that the best planning strategy to both improve PM absorption and maintain the species diversity of street trees is to increase the number of street trees while employing a mixture of the various species. Additionally, we found that it is necessary to plant both evergreen trees and broadleaf trees so as to cope with PM pollution in winter, when PM concentrations tend to be higher.

This study bears limitations in that it included only trees and did not consider shrubs or herbs. Furthermore, because of the inherent data limitation, the study was unable to consider more diverse tree species. Nevertheless, this study exhibits the risks of losing valuable environmental benefits when pursuing only a specific single goal with respect to street tree planting. Furthermore, the study provided useful knowledge for street tree planers and which strategies they should take according to the situation. Moreover, the system dynamics model developed in this study can be leveraged as a tool for supporting their decision-making.

## Figures and Tables

**Table 1:** Average annual PM absorption by trees [10,42].

Tree Species	Annual PM Absorption	Unit
*Zelkova serrata*	66.6	g/tree/Year
*Prunus yedoensis*	45.3	g/tree/Year
*Pinus densiflora*	24.2	g/tree/Year
other species ^1^	35.7	g/tree/Year

^1^ The data for other species refers to [42].

**Table 2:** Scenario settings.

Scenarios		Targeted Intensive Planting Ratio for Each Species	Initial Tree Composition				Initial Value of PM Absorption by Trees (g)
			Zelko	Prun	Pinus	Mix	
Baseline Scenario		N/A	112	70	0	26	963
Replace-only scenarios	Rep_only_Zelko	Plant *Zelkova serrata* 70% (~up to 90%) priority	112	70	0	26	963
	Rep_only_Prun	Plant *Prunus yedoensis* 70% (~up to 90%) priority					
	Rep_only_Pinus	Plant *Pinus densiflora* 70% (~up to 90%) priority					
	Rep_only_Mix	Plant *Zelkova serrata* (30%), *Prunus yedoensis* (30%), *Pinus densiflora* (30%), and others (10%)					
Additional Tree Planting scenarios	Plant_more_Zelko	Plant *Zelkova serrata* 70% priority	292	70	0	26	1962
	Plant_more_Prun	Plant *Prunus yedoensis* 70% priority	112	250	0	26	1643
	Plant_more_Pinus	Plant *Pinus densiflora* 70% priority	112	70	180	26	1326
	Plant_more_Mix	Plant *Zelkova serrata* (30%), *Prunus yedoensis* (30%), *Pinus densiflora* (30%), and others (10%)	172	130	60	26	1644

**Table 3:** The results of scenario analyses.

Criteria	Scenarios with the Best Result
Maximize PM absorption by street trees	1st: Plant_more_Zelko 2nd: Plant_more_Prun 3rd: Plant_more_Mix
Maximize species diversity among street trees	1st: Rep_only_Mix 2nd: Plant_more_Mix 3rd: Rep_only_Pinus
Increase both PM absorption by trees and species diversity among trees	1st and only: Plant_more_Mix
Increase PM abortion in winter	1st: Plant_more_Pinus 2nd: Plant_more_Mix 3rd: Plant_more_Zelko

## Data Availability

Data sharing not applicable.

## Supplementary Materials

The following supporting information can be downloaded at: https://www.mdpi.com/article/10.3390/ijerph19031625/s1, Table S1: Explain the input value of the variable.
