# Supplementary material for: Street Tree Planning to Improve Public Health and Ecosystem Resilience in Urban Areas: A Scenario Analysis Using a System Dynamics Model
Source: Int J Environ Res Public Health. 2022 Jan 31;19(3):1625. doi: 10.3390/ijerph19031625 (PMC8834800; doi:10.3390/ijerph19031625)
Supplement: Supplementary file 1 [file ijerph-19-01625-s001.zip › ijerph-1544159-supplementary.pdf]

Supplementary Material

1. Model Structure

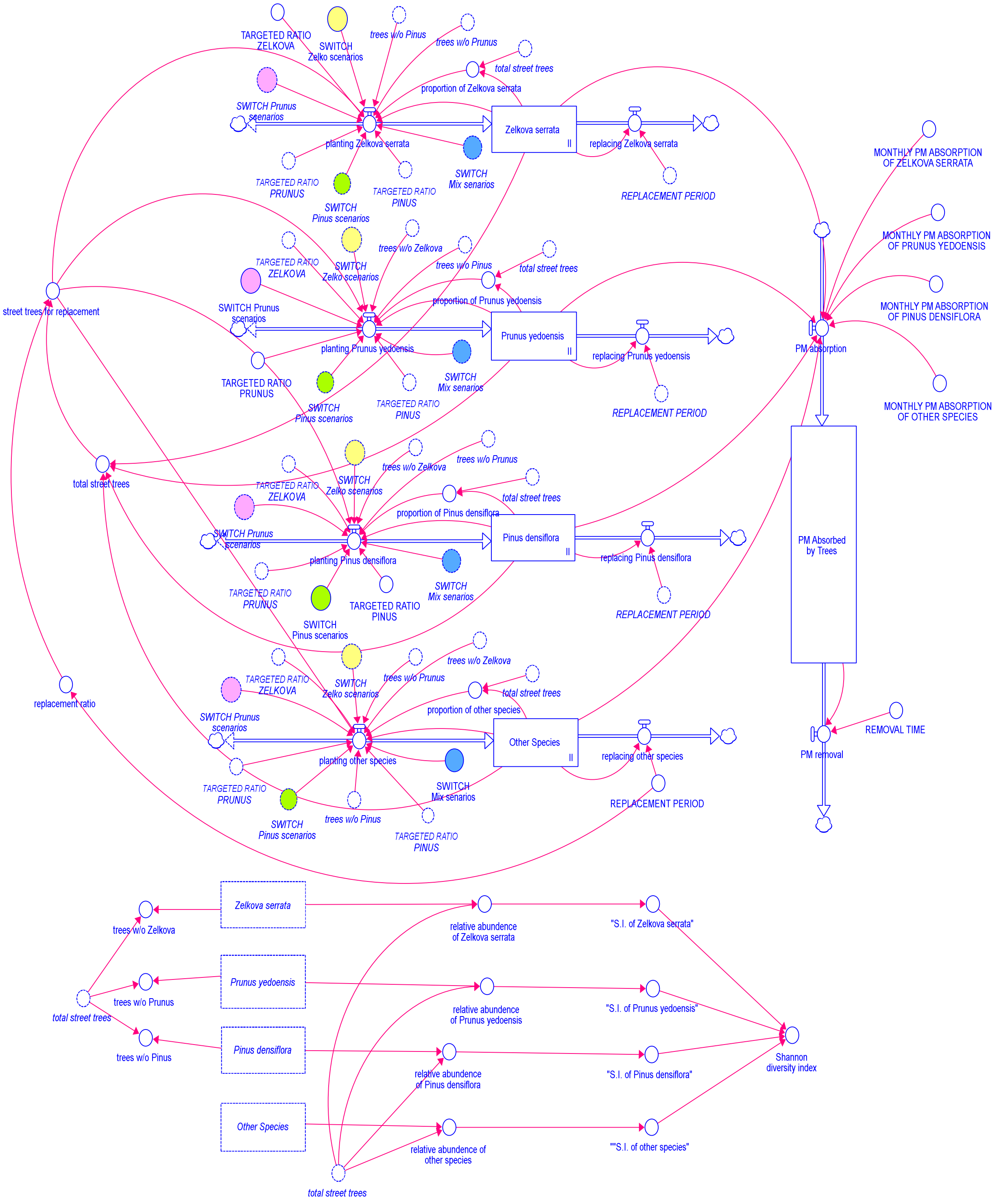

Figure S1. Entire model structure

2. Model Equations

Table S1. Model Equations

Variable type	Variable name	Equation	Units
	Zelkova_serrata(t)	Zelkova_serrata(t - dt) + (planting_Zelkova_serrata - replacing_Zelkova_serrata) * dt	tree
	Prunus_yedoensis(t)	Prunus_yedoensis(t - dt) + (planting_Prunus_yedoensis - replacing_Prunus_yedoensis) * dt	tree
	Pinus_densiflora(t)	Pinus_densiflora(t - dt) + (planting_Pinus_densiflora - replacing_Pinus_densiflora) * dt	tree
	Other_Species(t)	Other_Species(t - dt) + (planting_other_species - replacing_other_species) * dt	tree
	PM_Absorbed_by_Trees(t)	PM_Absorbed_by_Trees(t - dt) + (PM_absorption - PM_removal) * dt	g
	planting_Zelkova_serrata	SWITCH_Zelko_scenarios*TARGETED_RATIO_ZELKOVA*street_trees_for_replacement<br><br>+SWITCH_Prunus_scenarios*(1-TARGETED_RATIO_PRUNUS)*street_trees_for_replacement*(Zelkova_serrata/"trees_w/o_Prunus")<br><br>+SWITCH_Pinus_scenarios*(1-TARGETED_RATIO_PINUS)*street_trees_for_replacement*(Zelkova_serrata/"trees_w/o_Pinus")<br><br>+SWITCH_Mix_senarios*TARGETED_RATIO_ZELKOVA*street_trees_for_replacement<br><br>+(1-SWITCH_Zelko_scenarios)*(1-SWITCH_Prunus_scenarios)*(1-SWITCH_Pinus_scenarios)*(1-SWITCH_Mix_senarios)*street_trees_for_replacement*proportion_of_Zelkova_serrata	Tree /Months
	planting_Prunus_yedoensis	SWITCH_Zelko_scenarios*(1-TARGETED_RATIO_ZELKOVA)*street_trees_for_replacement*(Prunus_yedoensis/"trees_w/o_Zelkova")<br><br>+SWITCH_Prunus_scenarios*TARGETED_RATIO_PRUNUS*street_trees_for_replacement<br><br>+SWITCH_Pinus_scenarios*(1-TARGETED_RATIO_PINUS)*street_trees_for_replacement*(Prunus_yedoensis/"trees_w/o_Pinus")<br><br>+SWITCH_Mix_senarios*TARGETED_RATIO_PRUNUS*street_trees_for_replacement<br><br>+(1-SWITCH_Zelko_scenarios)*(1-SWITCH_Prunus_scenarios)*(1-SWITCH_Pinus_scenarios)*(1-SWITCH_Mix_senarios)*street_trees_for_replacement*proportion_of_Prunus_yedoensis	Tree /Months
	planting_Pinus_densiflora	SWITCH_Zelko_scenarios*(1-TARGETED_RATIO_ZELKOVA)*street_trees_for_replacement*(Pinus_densiflora/"trees_w/o_Zelkova")<br><br>+SWITCH_Prunus_scenarios*(1-TARGETED_RATIO_PRUNUS)*street_trees_for_replacement*(Pinus_densiflora/"trees_w/o_Prunus")<br><br>+SWITCH_Pinus_scenarios*TARGETED_RATIO_PINUS *street_trees_for_replacement	Tree /Months

		+SWITCH_Mix_senarios*TARGETED_RATIO_PINUS *street_trees_for_replacement<br><br>+(1-SWITCH_Zelko_scenarios)*(1-SWITCH_Prunus_scenarios)*(1-SWITCH_Pinus_scenarios)*(1-SWITCH_Mix_senarios)*street_trees_for_replacement*proportion_of_Pinus_densiflora	
	planting_other_species	SWITCH_Zelko_scenarios*(1-TARGETED_RATIO_ZELKOVA)*street_trees_for_replacement*(Other_Species/"trees_w/o_Zelkova")<br><br>+SWITCH_Prunus_scenarios*(1-TARGETED_RATIO_PRUNUS)*street_trees_for_replacement*(Other_Species/"trees_w/o_Prunus")<br><br>+SWITCH_Pinus_scenarios*(1-TARGETED_RATIO_PINUS)*street_trees_for_replacement*(Other_Species/"trees_w/o_Pinus")<br><br>+SWITCH_Mix_senarios*(1-TARGETED_RATIO_ZELKOVA-TARGETED_RATIO_PRUNUS-TARGETED_RATIO_PINUS)*street_trees_for_replacement<br><br>+(1-SWITCH_Zelko_scenarios)*(1-SWITCH_Prunus_scenarios)*(1-SWITCH_Pinus_scenarios)*(1-SWITCH_Mix_senarios)*street_trees_for_replacement*proportion_of_other_species	Tree /Months
	replacing_Zelkova_serrata	Zelkova_serrata/REPLACEMENT_PERIOD	Tree /Months
	replacing_Prunus_yedoensis	Prunus_yedoensis/REPLACEMENT_PERIOD	Tree /Months
	replacing_Pinus_densiflora	Pinus_densiflora/REPLACEMENT_PERIOD	Tree /Months
	replacing_other_species	Other_Species/REPLACEMENT_PERIOD	Tree /Months
	PM_absorption	(Zelkova_serrata*MONTHLY_PM_ABSORPTION_OF_ZELKOVA_SERRATA)<br>+(Prunus_yedoensis*MONTHLY_PM_ABSORPTION_OF_PRUNUS_YEDOENSIS)<br>+(Pinus_densiflora*MONTHLY_PM_ABSORPTION_OF_PINUS_DENSIFLORA)<br>+(Other_Species*MONTHLY_PM_ABSORPTION_OF_OTHER_SPECIES)	g/Months
	PM_removal	PM_Absorbed_by_Trees/REMOVAL_TIME	g/Months
	total_street_trees	Zelkova_serrata + Prunus_yedoensis + Pinus_densiflora + Other_Species	tree
	"trees_w/o_Zelkova"	total_street_trees-Zelkova_serrata	tree
	"trees_w/o_Prunus"	total_street_trees-Prunus_yedoensis	tree
	"trees_w/o_Pinus"	total_street_trees-Pinus_densiflora	tree
	proportion_of_ Zelkova_serrata	Zelkova_serrata/total_street_trees	1
	proportion_of_ Prunus_yedoensis	Prunus_yedoensis/total_street_trees	1
	proportion_of_ Pinus_densiflora	Pinus_densiflora/total_street_trees	1
	proportion_of_other_species	Other_Species/total_street_trees	1
	relative_abundence_of_ Zelkova_serrata	Zelkova_serrata/total_street_trees	1
	relative_abundence_of_ Prunus_yedoensis	Prunus_yedoensis/total_street_trees	1

○	relative_abundence_of_Pinus_densiflora	Pinus_densiflora/total_street_trees	1
○	relative_abundence_of_other_species	Other_Species/total_street_trees	1
○	"S.I._of_Zelkova_serrata"	-relative_abundence_of_Zelkova_serrata*LN(relative_abundence_of_Zelkova_serrata)	1
○	"S.I._of_Prunus_yedoensis"	-relative_abundence_of_Prunus_yedoensis*LN(relative_abundence_of_Prunus_yedoensis)	1
○	"S.I._of_Pinus_densiflora"	IF(relative_abundence_of_Pinus_densiflora=0) THEN 0 ELSE (-relative_abundence_of_Pinus_densiflora*LN(relative_abundence_of_Pinus_densiflora))	1
○	"S.I._of_other_species"	IF(relative_abundence_of_other_species=0) THEN 0 ELSE (-relative_abundence_of_other_species*LN(relative_abundence_of_other_species))	1
○	Shannon_diversity_index	"S.I._of_Zelkova_serrata"+"S.I._of_Prunus_yedoensis"+"S.I._of_Pinus_densiflora"*"\ "S.I._of_other_species"	1
○	replacement_ratio	1/REPLACEMENT_PERIOD	1/Month
○	street_trees_for_replacement	replacement_ratio*total_street_trees	Tree /Months
○	REMOVAL_TIME	1	Months
○	REPLACEMENT_PERIOD	20*12	Months

3. Input Values of Parameters

3.1 PM Absorption of Trees

Table S2. Model equations for PM absorption of trees

Parameter name	Input values	Units
MONTHLY_PM_ABSORPTION_OF_ZELKOVA_SERRATA	66.6/12	g/tree/month
MONTHLY_PM_ABSORPTION_OF_PRUNUS_YEDOENSIS	45.3/12	g/tree/month
MONTHLY_PM_ABSORPTION_OF_PINUS_DENSIFLORA	24.2/12	g/tree/Month
MONTHLY_PM_ABSORPTION_OF_OTHER_SPECIES	35.7/12	g/tree/month

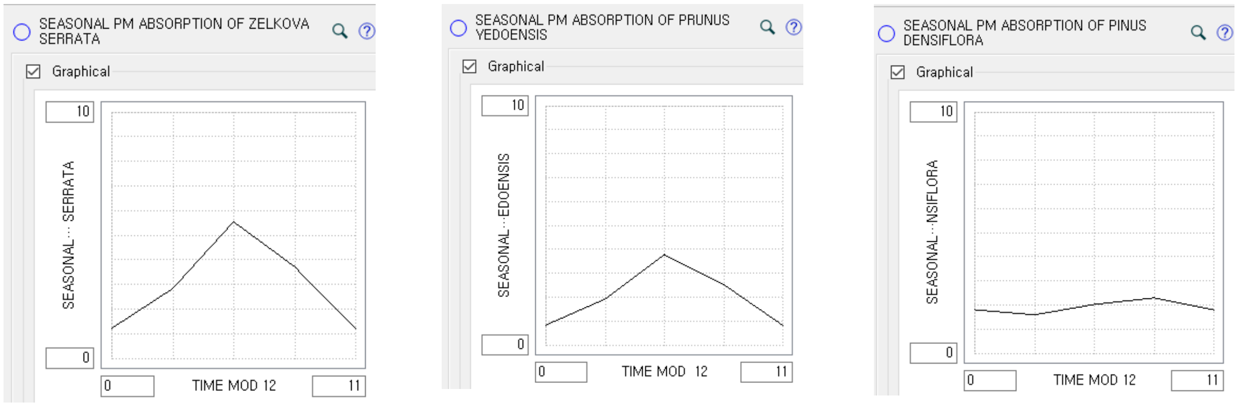

(a) (b) (c)

Figure S2. Model equations for seasonal PM absorption of trees: (a) *Zelkova serrata*; (b) *Prunus yedoensis*; (c) *Pinus densiflora*.

3.2 Scenario analysis

Table S3. Model equations for scenario analysis

Scenario	Parameter name	Input values	Units
Baseline	SWITCH_Zelko_scenarios	0	1
	SWITCH_Prunus_scenarios	0	1
	SWITCH_Pinus_scenarios	0	1
	SWITCH_Mix_senarios	0	1
	TARGETED_RATIO_ZELKOVA	0	1
	TARGETED_RATIO_PRUNUS	0	1
	TARGETED_RATIO_PINUS	0	1
	Initial value of Zelkova serrata	112	tree
	Initial value of Prunus yedoensis	70	tree
	Initial value of Pinus densiflora	0	tree
	Initial value of Other species	26	tree
	Initial value of PM absorption by trees	963	g
Rep_only_Zelko	SWITCH_Zelko_scenarios	1	1
	SWITCH_Prunus_scenarios	0	1
	SWITCH_Pinus_scenarios	0	1
	SWITCH_Mix_senarios	0	1
	TARGETED_RATIO_ZELKOVA	0.7	1
	TARGETED_RATIO_PRUNUS	0	1
	TARGETED_RATIO_PINUS	0	1
	Initial value of Zelkova serrata	112	tree
	Initial value of Prunus yedoensis	70	tree
	Initial value of Pinus densiflora	0	tree
	Initial value of Other species	26	tree
	Initial value of PM absorption by trees	963	g
Rep_only_Prun	SWITCH_Zelko_scenarios	0	1
	SWITCH_Prunus_scenarios	1	1
	SWITCH_Pinus_scenarios	0	1
	SWITCH_Mix_senarios	0	1
	TARGETED_RATIO_ZELKOVA	0	1
	TARGETED_RATIO_PRUNUS	0.7	1
	TARGETED_RATIO_PINUS	0	1
	Initial value of Zelkova serrata	112	tree
	Initial value of Prunus yedoensis	70	tree
	Initial value of Pinus densiflora	0	tree
	Initial value of Other species	26	tree
	Initial value of PM absorption by trees	963	g
Rep_only_Pinus	SWITCH_Zelko_scenarios	0	1
	SWITCH_Prunus_scenarios	0	1
	SWITCH_Pinus_scenarios	1	1
	SWITCH_Mix_senarios	0	1
	TARGETED_RATIO_ZELKOVA	0	1
	TARGETED_RATIO_PRUNUS	0	1
	TARGETED_RATIO_PINUS	0.7	1
	Initial value of Zelkova serrata	112	tree
	Initial value of Prunus yedoensis	70	tree
	Initial value of Pinus densiflora	0	tree
	Initial value of Other species	26	tree

	Initial value of PM absorption by trees	963	g
Rep_only_Mix	SWITCH_Zelko_scenarios	0	1
	SWITCH_Prunus_scenarios	0	1
	SWITCH_Pinus_scenarios	0	1
	SWITCH_Mix_senarios	1	1
	TARGETED_RATIO_ZELKOVA	0.3	1
	TARGETED_RATIO_PRUNUS	0.3	1
	TARGETED_RATIO_PINUS	0.3	1
	Initial value of Zelkova serrata	112	tree
	Initial value of Prunus yedoensis	70	tree
	Initial value of Pinus densiflora	0	tree
	Initial value of Other species	26	tree
	Initial value of PM absorption by trees	963	g
Plant_more_Zelko	SWITCH_Zelko_scenarios	1	1
	SWITCH_Prunus_scenarios	0	1
	SWITCH_Pinus_scenarios	0	1
	SWITCH_Mix_senarios	0	1
	TARGETED_RATIO_ZELKOVA	0.7	1
	TARGETED_RATIO_PRUNUS	0	1
	TARGETED_RATIO_PINUS	0	1
	Initial value of Zelkova serrata	292	tree
	Initial value of Prunus yedoensis	70	tree
	Initial value of Pinus densiflora	0	tree
	Initial value of Other species	26	tree
	Initial value of PM absorption by trees	1962	g
Plant_more_Prun	SWITCH_Zelko_scenarios	0	1
	SWITCH_Prunus_scenarios	1	1
	SWITCH_Pinus_scenarios	0	1
	SWITCH_Mix_senarios	0	1
	TARGETED_RATIO_ZELKOVA	0	1
	TARGETED_RATIO_PRUNUS	0.7	1
	TARGETED_RATIO_PINUS	0	1
	Initial value of Zelkova serrata	112	tree
	Initial value of Prunus yedoensis	250	tree
	Initial value of Pinus densiflora	0	tree
	Initial value of Other species	26	tree
	Initial value of PM absorption by trees	1643	g
Plant_more_Pinus	SWITCH_Zelko_scenarios	0	1
	SWITCH_Prunus_scenarios	0	1
	SWITCH_Pinus_scenarios	1	1
	SWITCH_Mix_senarios	0	1
	TARGETED_RATIO_ZELKOVA	0	1
	TARGETED_RATIO_PRUNUS	0	1
	TARGETED_RATIO_PINUS	0.7	1
	Initial value of Zelkova serrata	112	tree
	Initial value of Prunus yedoensis	70	tree
	Initial value of Pinus densiflora	180	tree
	Initial value of Other species	26	tree
	Initial value of PM absorption by trees	1326	g
Plant_more_Mix	SWITCH_Zelko_scenarios	0	1
	SWITCH_Prunus_scenarios	0	1
	SWITCH_Pinus_scenarios	0	1

SWITCH_Mix_senarios	1	1
TARGETED_RATIO_ZELKOVA	0.3	1
TARGETED_RATIO_PRUNUS	0.3	1
TARGETED_RATIO_PINUS	0.3	1
Initial value of Zelkova serrata	172	tree
Initial value of Prunus yedoensis	130	tree
Initial value of Pinus densiflora	60	tree
Initial value of Other species	26	tree
Initial value of PM absorption by trees	1644	g
